# Controllability Modulates the Anticipatory Response in the Human Ventromedial Prefrontal Cortex

**DOI:** 10.3389/fpsyg.2012.00557

**Published:** 2012-12-14

**Authors:** Deborah L. Kerr, Donald G. McLaren, Robin M. Mathy, Jack B. Nitschke

**Affiliations:** ^1^Waisman Center for Brain Imaging and Behavior, University of Wisconsin-MadisonMadison, WI, USA; ^2^Department of Psychology, University of Wisconsin-MadisonMadison, WI, USA; ^3^Department of Neurology, Massachusetts General HospitalBoston, MA, USA; ^4^Athinoula A. Martinos Center for Biomedical Imaging, Massachusetts General HospitalCharlestown, MA, USA; ^5^Geriatric Research, Education and Clinical Center, Edith Nourse Rogers Memorial VA Medical CenterBedford, MA, USA; ^6^Harvard Medical SchoolBoston, MA, USA; ^7^Kellogg College, University of OxfordOxford, UK; ^8^Wolfson College, University of CambridgeCambridge, UK; ^9^Department of Social Science, Central Oregon Community CollegeBend, OR, USA; ^10^Department of Psychiatry, University of Wisconsin-MadisonMadison, WI, USA

**Keywords:** controllability, anticipation, vmPFC, amygdala, fMRI, PPI, phobia

## Abstract

Research has consistently shown that control is critical to psychological functioning, with perceived lack of control considered to play a crucial role in the manifestation of symptoms in psychiatric disorders. In a model of behavioral control based on non-human animal work, Maier et al. (2006) posited that the presence of control activates areas of the ventromedial prefrontal cortex (vmPFC), which in turn inhibit the normative stress response in the dorsal raphe nucleus and amygdala. To test Maier’s model in humans, we investigated the effects of control over potent aversive stimuli by presenting video clips of snakes to 21 snake phobics who were otherwise healthy with no comorbid psychopathologies. Based on prior research documenting that disrupted neural processing during the anticipation of adverse events can be influenced by different forms of cognitive processing such as perceptions of control, analyses focused on the anticipatory activity preceding the videos. We found that phobics exhibited greater vmPFC activity during the anticipation of snake videos when they had control over whether the videos were presented as compared to when they had no control over the presentation of the videos. In addition, observed functional connectivity between the vmPFC and the amygdala is consistent with previous work documenting vmPFC inhibition of the amygdala. Our results provide evidence to support the extension of Maier’s model of behavioral control to include anticipatory function in humans.

## Introduction

Emotion and cognition interact in numerous ways that affect psychopathology. Importantly, resilience has the potential to significantly mitigate human suffering related to psychopathology (Garmezy, 1971; Masten, 2001, 2011; Casey, 2011). The capacity to perceive control, to identify controllable situations, and to exert effortful control is involved in the complex process leading to resilience (Staudinger et al., 1995; Chorpita and Barlow, 1998; Kumpfer, 1999; Maier et al., 2006; Eisenberg and Sulik, 2012). Moreover, perceived control can dampen emotional responses to aversive events, which in turn would mitigate any impairing effects of emotion on cognition. Indeed, controllability has been a core concept in empirical and theoretical work on psychopathology (e.g., Freud, 1936; Mandler and Watson, 1966; Barlow, 2002) and resilience (Kumpfer, 1999; Zimmerman et al., 1999; Bandura et al., 2003; Yi et al., 2005; Rutter, 2008).

Although multiple aspects of controllability are distinctively human (Abramson et al., 1978; Bandura, 1989; Bandura et al., 2003), research with non-human animals has provided important insights about the mechanisms involved in behavioral control. Influential work in non-human animals has demonstrated differential behavioral phenotypes in response to electrical shock dependent on the animal’s perception that it can or cannot escape/avoid the shock (Overmier and Seligman, 1967; Seligman and Maier, 1967; Seligman et al., 1968; Seligman and Beagley, 1975; Seligman et al., 1975). The inescapable response phenomenon, termed *learned helplessness* (Seligman et al., 1975), has generated numerous lines of research. The extension of Seligman’s learned helplessness model to humans (Hiroto and Seligman, 1975) required refinement precisely because humans are a meaning-making species and attribute helplessness to a cause, whether “stable or unstable, global or specific, and internal or external” (Abramson et al., 1978, p. 49). This reformulation made Bandura’s (1969, 1977, 1986, 1997) social learning and social cognitive theories essential for an understanding of causal attributions, including controllability, when humans perceive themselves to be helpless under adverse or potentially adverse circumstances. This led to demonstrations that affective self-regulation (which may be perceived as internalized/implicit control) is essential to positive psychological adaptation (Bandura et al., 2003; Eisenberg and Sulik, 2012). Thus, any cogent extension of non-human animal research to human neurobiology must acknowledge the role of social cognition/self-efficacy in making causal attributions about perceived helplessness in humans.

Research on external control in non-human animals has uncovered many of the neurobiological mechanisms involved (Weiss, 1991; Maier et al., 2006). This provides insights into which neuroanatomical structures should be investigated in explorations of control in humans. In particular, increased serotonergic response in the dorsal raphe nucleus (DRN) is necessary for learned helplessness (Maier et al., 1993, 1995); input to the DRN is almost exclusively from the infralimbic and prelimbic areas of the ventromedial prefrontal cortex (vmPFC; Jankowski and Sesack, 2004; Gabbott et al., 2005); and activation of the vmPFC decreases the learned helplessness response, whereas inhibition of the vmPFC increases the learned helplessness response (Amat et al., 2005, 2006). Furthermore, activity in the vmPFC inhibits the normative stress response in relevant midbrain, limbic, brainstem, and cortical areas such as the DRN (Amat et al., 2005) and amygdala (Maier et al., 2006). These findings led Maier et al. (2006) to posit that the presence of control and top-down feedback by the vmPFC are critical for resilient behavior.

Research extending these findings to the human vmPFC has been minimal. Two fMRI studies on pain have demonstrated alterations in the neural response to pain when subjects perceive that they have control over the duration of the painful stimulus (Salomons et al., 2004; Wiech et al., 2006). Of particular importance for investigating controllability in humans is anticipatory function, as the ability to anticipate threatening situations is critical to the survival of any organism. The capacity of anticipating the future is further highlighted in Bandura’s views on self-efficacy and resilience, with an emphasis on predicting beneficial as well as aversive consequences, setting goals, and planning actions to arrive at desired outcomes (Bandura, 1989; Bandura et al., 2003). In humans, excessive anticipation of negative events has been shown to be maladaptive and to contribute to psychiatric disorders (Mackiewicz et al., 2006; Nitschke et al., 2006, 2009; Straube et al., 2007; Sarinopoulos et al., 2010). Therefore, we posit that the debilitating effects of anxious anticipation in psychiatric disorders may be the result of, or compounded by, the perceived uncontrollability of the event. Furthering our understanding of the relationships between control and anticipation are of paramount importance in human research and the development of therapeutic interventions that can increase resilience.

To investigate the neural underpinnings of controllability in humans, we designed a study that robustly elicited aversion in a scenario that provided a strong test of control. A sample of 21 snake phobics who were otherwise healthy with no comorbid psychopathologies viewed video clips of moving snakes. On half the trials, an anticipatory cue indicated that they could avert the video presentation (controllable) if they responded quickly enough to a target. For the other half of the trials, the cue indicated that their response times to the target had no impact on the video presentation (uncontrollable). The controllable condition is tightly linked to the concept of perceived control, which has been identified as central to resilience research.

We hypothesized heightened vmPFC activity during the anticipation of controllable snake (cS) videos and increased functional connectivity of the vmPFC with the amygdala (Carlsson et al., 2004; Larson et al., 2006; Maier et al., 2006; Straube et al., 2006; also reviewed in Etkin and Wager, 2007). In the current study, connectivity was operationalized using a new method of context-dependent connectivity (McLaren et al., 2012) building on psychophysiological interactions (PPI; Friston et al., 1997; Gitelman et al., 2003). Support for these hypotheses would demonstrate that Maier et al.’s (2006) model of behavioral control over responses to stress and aversion extends to anticipatory responses in humans, which has important consequences on how emotion modulates cognition.

## Materials and Methods

### Participants

Twenty-one snake phobic participants (17 females, mean age 21.8, range 18–46), without any comorbid psychopathologies, were recruited to this study from the University of Wisconsin at Madison undergraduate population and surrounding community. All participants were right-handed and neurologically normal. Participants were diagnosed with specific phobia (of snakes) using the Structured Clinical Interview for the DSM-IV (SCID; First et al., 2002) and had never taken any prescribed psychotropic medications or participated in behavioral therapy. Participants provided informed written consent and were paid for their participation. The study was approved by the University of Wisconsin-Madison Health Sciences Institutional Review Board in accordance with the Declaration of Helsinki.

### Event-related experimental paradigm

**Figure 1:**
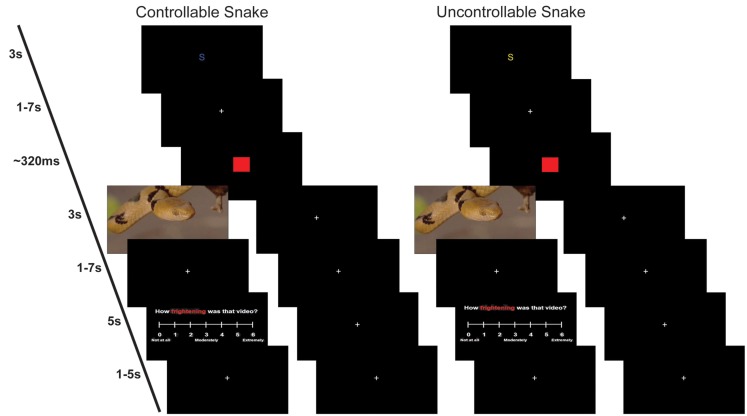
**Example trials**. Left: a controllable snake trial where participant did not press the button fast enough and saw a video of a snake (left branch) or where participant pressed the button fast enough and did not see a video of a snake (right branch). Right: an uncontrollable snake trial where participant saw a video of a snake (left branch) or where participant did not see a video of a snake (right branch). The cue, presented for 3 s, was either yellow indicating an uncontrollable trial or blue indicating a control trial. The letter S indicated the potential video contained a snake. Following the cue, a fixation cross appeared for 1–7 s. We modeled the anticipation period as the time of the cue plus fixation cross. Following the fixation cross, a red square would appear, and the participant pressed a button as quickly as possible. Depending on the trial type and speed of the response, either a video or fixation cross would appear for 3 s. This was followed by another fixation cross for 1–7 s. If the participant saw a video, then they had 5 s to answer a rating question using a Likert scale. The scale was followed by a final fixation cross for 1–5 s before the onset of the next trial.

Each trial began with an anticipation epoch containing a colored letter cue signal plus a variable delay period (Figure 1). The S cue indicated that a phobogenic stimulus of a snake video clip (e.g., one snake crawling) might follow. The F cue indicated that a neutral stimulus of a fish video clip (e.g., one fish swimming) might follow. Each video was equalized for several physical attributes (brightness, contrast, scene complexity, and movement). Videos were selected from 90 videos (30 snake videos, 27 fish videos, and 33 disgust videos) that were rated by 19 adults (7 females) with a median age of 25.5 (range 19–58). Participants rated each video for: valence, arousal, fear, disgust, certainty (of viewed content), complexity, familiarity. The 78 videos in the present study were selected based on the stability of their ratings. Examples of each video type can be found in the Supplemental Material. Videos were presented to participants using the entire viewing area provided by a Silent Vision System (Avotec, Inc., Jensen Beach, FL, USA).

The anticipation epoch was further divided into a perceived controllable and a perceived uncontrollable condition. A blue cue indicated the participant had control over whether the video would be seen or not (controllable trial), while a yellow cue indicated the participant had no control over whether the video would be seen or not (uncontrollable trial). After the variable delay period, a target red square was presented that the participant was told to press a button to as quickly as possible. For all trials, the instructions were the same: “Press the button as fast as possible when the target red square appears.” The target was followed by either a video clip or a fixation cross. When a participant had a controllable trial they were informed that if they responded fast enough to the red target square, they would see a fixation cross instead of the video; however, if they were not fast enough, they would see a video. When a participant had an uncontrollable trial, they were informed a video clip would follow on half the trials and a fixation cross would follow on the other half of the trials. To ensure that participants were only able to avoid the videos on approximately 50% of the control trials, the target presentation time was adjusted on a trial-by-trial basis using DMDX software (Jonathan Forster, University of Arizona). If a participant failed to respond fast enough to avoid the video on one trial, the target presentation of the subsequent trial was lengthened by 17–149 ms. Conversely, if a participant responded fast enough to avoid the video, the target presentation of the subsequent trial was shortened by 16–100 ms. Videos were presented for 3 s followed by a variable delay period. Following the presentations of videos, one Likert online rating about the nature of the stimulus was collected per trial: (a) valence; (b) arousal; (c) disgust; and (d) fear. Participants had 5 s to make their rating, which was then followed by a variable intertrial interval. Colors and rating questions were counterbalanced. In summary, this manuscript focuses on four conditions: (1) cS, anticipation epoch that precedes a potential snake video where the participant can avoid the video; (2) cF, anticipation epoch that precedes a potential fish video where the participant can avoid the video; (3) uS, anticipation epoch that precedes a potential snake video where participant response does not affect video presentation; (4) uF, anticipation epoch that precedes a potential fish video where participant response does not affect video presentation.

### Data acquisition

All participants underwent fMRI scanning during four runs of the experimental paradigm consisting of 132 trials. The breakdown of trial types was as follows: 22 controllable snake (cS), 22 uncontrollable snake (uS), 22 controllable fish (cF), and 22 uncontrollable fish (uF). Two weeks prior to fMRI scanning, participants underwent a mock scan during which they viewed an abbreviated version of the experimental paradigm using different videos from those used in the actual fMRI scan. Of note, disgust trials were also included in the paradigm, with the D cue indicating that a disgust video clip (e.g., moving maggots, vomiting) might follow. The corresponding 22 controllable and 22 uncontrollable disgust trial types were modeled at the first-level, but not utilized in the group analyses. Disgust trials were not analyzed at the group level because participants’ self-reports during debriefing immediately following the fMRI scan revealed mixed responses on how they viewed the disgust trials, including morbid fascination and excited curiosity. Moreover, the behavioral responses to the target did not show the expected pattern of reduced reaction times to controllable than uncontrollable aversive stimuli. Thus, the disgust trials were excluded because they were not universally aversive.

A 3.0 Tesla GE SIGNA Scanner (Milwaukee, WI, USA) with a quadrature birdcage head coil was used to collect anatomical and functional images. Two sagittal GRE field maps were acquired in order to correct warping of the experimental echo planar imaging (EPI) scans around tissue-air interfaces such as the forehead, the brainstem, and the sinuses (Cusack et al., 2003), with the following parameters: repetition time (TR) = 700 ms, echo time (TE)1/TE2 = 7/10 ms, field-of-view (FOV) = 24 cm, flip angle = 60°, number of excitations (NEX) = 1, matrix = 256 × 128, 30 sagittal slices of 4.0 mm, and a gap of 1.0 mm. Functional data was collected using a sagittal, T2*-weighted, blood oxygen-level dependant (BOLD) EPI sequence with the following parameters: TR = 2 s, TE = 30 ms, FOV = 24 cm, flip angle = 90°, NEX = 1, matrix = 64 × 64, voxel size = 3.75 mm, 30 slices, slice thickness = 4.0 mm, gap = 1.0 mm. Each of the four functional runs was 267 TRs. Finally, we collected a 3D T1-weighted inversion-recovery fast gradient echo sequence with the following parameters: TR = 8.9 ms, TE = 1.8 ms, inversion time = 600 ms, FOV = 24 cm, flip angle = 10°, NEX = 1, matrix = 256 × 192, voxel size = 0.9375 mm, 124 slices, slice thickness = 1.2 mm.

### Image preprocessing

Images underwent the following preprocessing steps in Analysis of Functional Neuroimages (AFNI; Medical College of Wisconsin, WI, USA): (1) slice time correction; (2) motion correction; (3) field map correction; and (4) conversion to percent signal change.

### First-level task activation analyses

General linear models (GLM) in SPM8 (University College London, UK) were used to derive single subject activations. The design matrix was formed by separately convolving the canonical HRF from SPM8 with the presence of the stimuli for the anticipation, video, and rating periods. For anticipation, the presence was defined as the time between the cue onset and the target red square, which could be thought of as an epoch. The design matrix also included the motion parameters, a constant term, autoregressive (AR1) term, and a high-pass filter. In AFNI, the contrast images for each anticipation period (cS, uS, cF, uF) were spatially normalized to the Talairach atlas (Talairach and Tournoux, 1988) and resampled to 1 mm^3^ voxels.

### Second-level task activation analysis

Hypotheses examining differences in neural activation during the anticipation of controllable and uncontrollable snake and fish videos were tested using planned contrasts in AFNI. Significant clusters (*p* < 0.05) were defined as clusters contained at least 224 contiguous voxels with a *p*-value of *p* < 0.005 or at least 337 contiguous voxels with a *p*-value of *p* < 0.01 based on 3dClustSim (AFNI) within a controllability mask (“Nitschke_Lab” in the peak_nii toolbox)^1^.

### First-level psychophysiological interactions analyses

Percent signal change images were spatially normalized to the Talairach atlas (Talairach and Tournoux, 1988), resampled to 2 mm isotropic voxels, and smoothed with a 6 mm FWHM Gaussian filter. Generalized psychophysiological interactions (gPPI) were used to evaluate context-dependent connectivity, based on their improved sensitivity and specificity in detecting connectivity effects (McLaren et al., 2012), with the vmPFC. The vmPFC seed region was defined as a 3-mm radius sphere around the peak voxel of the cS minus uS contrast (Talairach: 5, 46, −7). We used the automated gPPI toolbox^2^ to estimate the PPI effects for each subject. This analysis was limited to the 12 participants who had full coverage in the region based on the mask generated by SPM8, rather than using variable seed regions for each participant. These 12 did not differ from the remaining nine participants for sex or age (all *p*s > 0.10).

### Second-level psychophysiological interactions analysis

Hypotheses examining functional connectivity via PPI were tested using one-sample *t*-tests of contrasts comparing two conditions (equivalent to paired *t*-tests). Significant clusters (*p* < 0.05) were defined as clusters contained at least 35 contiguous voxels with a *p*-value of *p* < 0.005 based on 3dClustSim within a controllability mask (“Nitschke_Lab” in the peak_nii toolbox, see text footnote 1).

## Results

### Behavioral results

As a manipulation check for perceived control, we tested whether reaction times differentiated the controllable and uncontrollable conditions. A 2 × 2 repeated-measures ANOVA examining controllability and stimulus revealed a significant interaction (*p* = 0.038). *Post hoc* paired *t*-test analyses of this interaction revealed that cS reaction times (mean = 458.77 ms; SEM = 24.89 ms) were significantly faster than uS reaction times (mean = 481.91 ms; SEM = 28.01 ms; *p* = 0.003). By contrast, there was not a significant difference between cF (mean = 488.15 ms; SEM = 27.30 ms) and uF reaction times (mean = 494.89 ms; SEM = 27.47 ms; *p* = 0.367). Additional comparisons revealed that cS reaction times were significantly faster than cF reaction times (*p* < 0.001) and that uS reaction times were significantly faster than uF (*p* = 0.035). These results show that controllability had a larger effect when the stimulus was aversive.

### fMRI activation results

**Figure 2:**
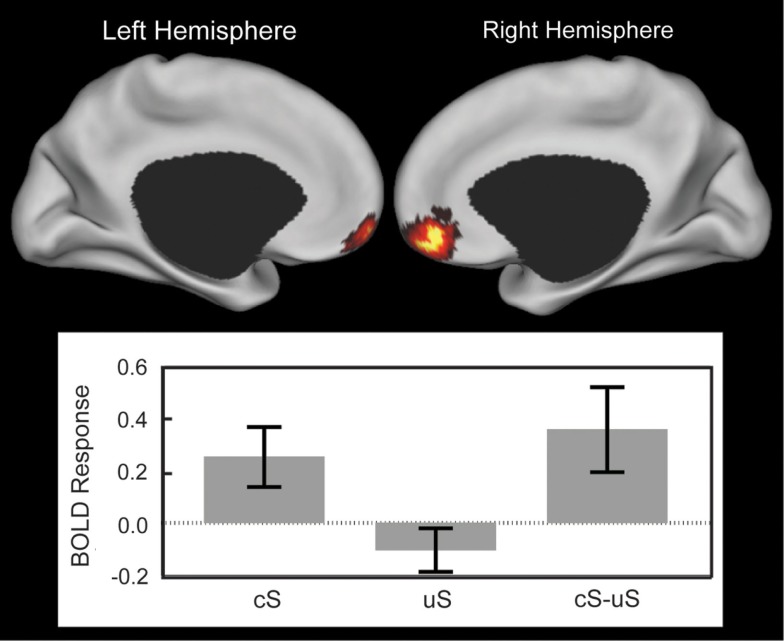
**Anticipatory ventromedial prefrontal cortex (vmPFC) response showing the effect of controllability**. Top: cortical surface renderings of the controllable snake (cS) minus uncontrollable snake (uS) contrast using multi-fiducial mapping in CARET with the strongest voxel within 2.5 mm of the surface (Van Essen, 2005). Results were thresholded at *p* < 0.05 cluster corrected. Brighter colors represent stronger effect or more overlap between the surfaces in the multi-fiducial map and can be interpreted as the most likely area of a strong effect. Bottom: the BOLD response in the cluster for cS, uS, and their difference. Error bars are SEM.

A paired *t*-test revealed greater anticipatory activation in the vmPFC for cS compared to uS (Figure 2, Tables 1 and 4). No other significant clusters were found for this comparison, nor were any significant clusters found in the opposite direction.

**Table 1:** **Anticipation of controllable snake videos > uncontrollable snake videos**.

Cluster size (mm^3^)	Peak location^a^	Talairach coordinates			Peak *t*-statistic	*p*-Value
		*x*	*y*	*z*	
389	vmPFC	−5	61	−10	4.613	<0.001
	vmPFC	5	46	−7	4.612	<0.001
	vmPFC	2	56	−11	3.796	<0.001

*^a^Table includes all significant peaks of activation that are more than 8 mm apart within significant clusters (*p* < 0.05 corrected). vmPFC, ventromedial prefrontal cortex*.

A paired *t*-test revealed greater anticipatory activation during the uF compared to cF in the posterior mid-cingulate cortex (pMCC), the right anterior insula, and the pons (Table 4). No significant clusters were found in either the vmPFC or amygdala, nor were any significant clusters found the opposite direction.

Within these regions, no interactions were found between controllability and stimuli, suggesting that the controllability effects were sub-threshold for non-aversive stimuli. Interestingly, there was an interaction in the left anterior insula (Table 4). *Post hoc* paired *t*-test analyses were conducted using values extracted from the left anterior insula cluster. These revealed that activity during cS was greater than that during uS (*p* = 0.015), whereas the activity during uF was greater than during cF (*p* = 0.004). Anticipatory activity during cS was greater than cF (*p* = 0.002), whereas the comparison for activity during uS compared uF was not significant (*p* = 0.624).

**Figure 3:**
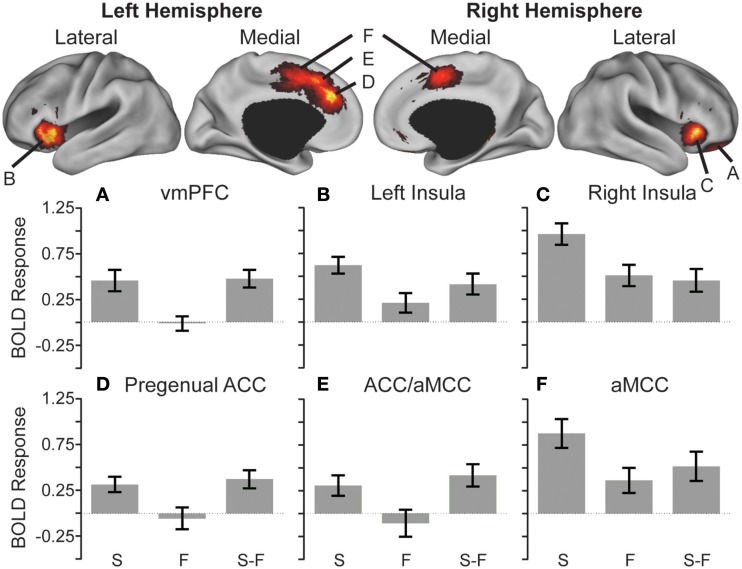
**Anticipatory activations showing the effect of stimulus**. Cortical surface renderings of snake (S) minus fish (F) contrast using multi-fiducial mapping in CARET with the strongest voxel within 2.5 mm of the surface (Van Essen, 2005). Results were thresholded at *p* < 0.05 cluster corrected. Bottom: heightened anticipatory activity reflected in greater activation in the ventromedial prefrontal cortex (vmPFC) **(A)**, bilateral anterior insula **(B,C)**, pregenual anterior cingulate cortex (ACC) **(D)**, regions spanning from the ACC to the anterior mid-cingulate cortex (aMCC) **(E)**, and bilateral aMCC **(F)** preceding snake videos compared to fish videos (S > F).

Valence effects collapsing across controllability were also assessed. A paired *t*-test revealed greater activity during the S compared to F in the vmPFC, the pregenual anterior cingulate cortex (pACC), a cluster spanning the anterior mid-cingulate cortex (aMCC), and ACC, a second cluster in the aMCC, bilateral anterior insula, and bilateral thalami (Figure 3, Tables 2 and 4). No significant effects were found in the opposite direction.

**Table 2:** **Anticipation of snake videos > fish videos**.

Cluster size (mm^3^)	Peak location^a^	Talairach coordinates			Peak *t*-statistic	*p*-Value
		*x*	*y*	*z*	
2727	Right thalamus	13	−9	13	5.534	<0.001
	Right thalamus	7	−27	6	5.339	<0.001
	Right thalamus	19	−14	17	5.335	<0.001
	Right thalamus	13	−20	16	4.738	<0.001
	Right thalamus	2	−6	8	3.841	<0.001
	Right thalamus	3	−19	9	3.418	0.001
	Right thalamus	17	−13	4	3.240	0.002
737	Left thalamus	−1	−7	8	4.082	<0.001
	Left thalamus	−10	−3	12	3.939	<0.001
	Left thalamus	−16	−11	17	3.608	<0.001
	Left thalamus	−16	−10	7	3.418	0.001
	Left thalamus	−6	−17	15	3.083	0.003
781	Left thalamus	−14	−25	12	3.714	<0.001
	Left thalamus	−15	−15	12	3.131	0.003
669	Left anterior insula	−29	24	4	4.249	<0.001
	Left anterior insula	−39	20	10	3.588	<0.001
	Left anterior insula	−35	11	3	3.574	0.001
	Left anterior insula	−30	17	−6	3.272	0.002
	Left anterior insula	−31	24	13	2.936	0.004
548	Right anterior insula	36	22	4	3.933	<0.001
495	vmPFC	18	37	−8	4.727	<0.001
	vmPFC	20	45	−8	4.265	<0.001
475	pACC	−4	32	26	4.717	<0.001
518	aMCC	−4	17	42	3.685	<0.001
	ACC	−5	26	37	2.777	0.006
729	aMCC	−6	2	45	3.504	0.001
	aMCC	6	3	47	3.090	0.003

*^a^Table includes all significant peaks of activation that are more than 8 mm apart within significant clusters (*p* < 0.05 corrected). ACC, anterior cingulate cortex; aMCC, anterior mid-cingulate cortex; pACC, pregenual anterior cingulate cortex; vmPFC, ventromedial prefrontal cortex*.

### fMRI context-dependent connectivity/gPPI results

A paired *t*-test revealed that the vmPFC contributed more to the activity in the pMCC during cS compared to uS (389 mm^3^; Table 4). There were no significant clusters where the connectivity was greater during uS compared to cS.

**Figure 4:**
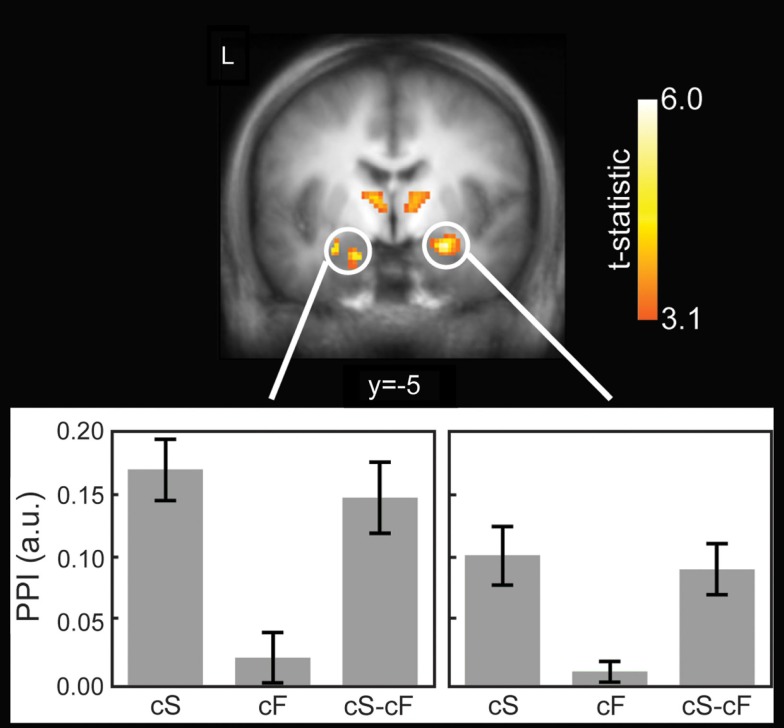
**Functional connectivity using generalized psychophysiological interactions (gPPI)**. Top: significantly greater connectivity during controllable snake (cS) compared to uncontrollable snake (uS) shown in a coronal slice through the amygdala and thalamus. Results were thresholded at *p* < 0.05 cluster corrected. Bottom: plots for the left and right amygdala clusters showing the PPI values. Error bars are SEM. a.u. = arbitrary units.

A paired *t*-test revealed that the vmPFC contributed more to the activity in the bilateral amygdala, pMCC, posterior cingulate, and bilateral thalami during cS compared to cF (Figure 4, Tables 3 and 4). There were no significant clusters where the connectivity was greater during cF compared to cS.

**Table 3:** **gPPI: functional connectivity between vmPFC and ROI during anticipation of controllable snake videos > controllable fish videos**.

Cluster size (mm^3^)	Peak location^a^	Talairach coordinates			Peak *t*-statistic	*p*-Value
		*x*	*y*	*z*	
984	Right ventral amygdala	23	−7	−12	6.028	<0.001
	Right extended amygdala	13	−11	−10	4.021	0.001
784	Left ventral amygdala	−19	−5	−16	4.773	<0.001
	Left extended amygdala	−27	−11	−10	4.699	<0.001
2832	Left pMCC	−13	−21	32	5.634	<0.001
	Left PCC	−11	−35	36	4.743	<0.001
504	Right PCC	15	−33	32	4.900	<0.001
3672	Left thalamus	−9	−9	10	4.695	<0.001
	Left thalamus	−13	−19	0	4.282	<0.001
	Right thalamus	11	−3	10	4.012	0.001
	Left thalamus	−13	−27	8	3.889	0.001
	Left thalamus	−17	−31	0	3.869	0.001
	Left thalamus	−23	−25	0	3.816	0.001
	Left thalamus	−13	−19	16	3.734	0.002
	Right thalamus	3	−11	6	3.529	0.002
472	Right thalamus	21	−23	0	3.926	0.001
	Right thalamus	15	−23	16	3.402	0.003
	Right thalamus	21	−31	10	3.147	0.005
312	Right PCC	5	−47	42	3.533	0.002

*^a^Table includes all significant peaks of activation that are more than 8 mm apart within significant clusters (*p* < 0.05 corrected). pMCC, posterior middle cingulate cortex; PCC, posterior cingulate cortex*.

**Table 4:** **Summary of results**.

Anatomical location	Paired *t*-tests											
	cS-uS		cF-uF		(cS-uS)-(cF-uF)		S-F		cS-uS		cS-cF	
	LH	RH	LH	RH	LH	RH	LH	RH	LH	RH	LH	RH
ACC							+					
aMCC							+	+				
pACC							+					
pMCC				−							+	
PCC									+		+	+
vmPFC	+	+					+					
Anterior insula				−	−		+	+				
Amygdala												
Ventral amygdala											+	+
Extended amygdala											+	+
Thalamus							+	+			+	+
Pons			−	−								

*NOTES: black, fMRI results; red, gPPI results; +, significant positive cluster; −, significant negative cluster; ACC, anterior cingulate cortex; aMCC, anterior mid-cingulate cortex; pACC, pregenual anterior cingulate cortex; PCC, posterior cingulate cortex; vmPFC, ventral medial prefrontal cortex; LH, left hemisphere; RH, right hemisphere*.

A paired *t*-test did not reveal any significant connectivity differences with the vmPFC between uS and uF.

## Discussion

This paper reports the first exploration of the neural basis for mediating the impact of perceived controllability on the anticipatory response to aversive stimuli. We found that in humans the vmPFC region is critical to behavioral control while anticipating aversive stimuli. Moreover, this area showed strong functional coupling with the amygdala, consistent with prior work implicating it in top-down regulation of the amygdala (Phelps et al., 2004; Urry et al., 2006; Johnstone et al., 2007; Maier and Watkins, 2010). This extends to humans the behavioral control model that Maier et al., 2006 based on their work with animals, the core of which emphasizes vmPFC regulation of the amygdala, and other brain areas that respond to stress. Based on our work and others results, we conclude that these brain regions are involved in mediating the impact of perceived control on emotional responses to adversity that can have enhancing or impairing effects on various domains of cognitive function.

Experiments dating back to the 1960s and 1970s have documented the effects of perceived control on behavioral responses (Seligman, 1975; Weiss, 1991; Barlow, 2002; Maier et al., 2006). These studies are pivotal because they demonstrated that: (1) there is a potential temporal dependence in learned helplessness (Overmier and Seligman, 1967; Seligman et al., 1975); (2) learned helplessness can be mitigated by prior escapable trials that induce perceived control (Seligman and Maier, 1967); and (3) learned helplessness can be reversed by showing that shocks are escapable (Seligman et al., 1968). These findings provided the impetus for investigating the neural basis for learned helplessness. Petty et al. (1994) demonstrated that learned helplessness correlated with serotonin levels in the vmPFC post-shock, but not basal pre-shock level, providing evidence that changes occur during the stressor. Subsequent studies demonstrated that vmPFC activity during inescapable shock correlated with later social exploration/escape behavior (Amat et al., 2005, 2006; Christianson et al., 2009) and that inhibiting vmPFC activity during, but not after, a forced swim test prevented the learned helplessness behavior the following day (Scopinho et al., 2010). Similar findings led Maier et al. (2006) to posit that the presence of control and its activation of the vmPFC are critical in determining behavior. In essence, the vmPFC modulates the stress response by top-down feedback.

The present study utilized a novel design to investigate the circuitry recruited by behavioral control in humans by exposing snake phobics to the very object on which their diagnosis is based. When they anticipated the snake videos, only the vmPFC showed a differential response between controllable and uncontrollable trials. Thus, the vmPFC has substantial potential to provide top-down feedback and aid in down regulation of the amygdala and stress-related responses. Consistent with this idea, we observed changes in connectivity with a number of brain regions, most notably the amygdala. In sum, the exact significance of the vmPFC is the implementation of perceived control in humans via its regulation of the stress response system.

Although the direction of the association between the vmPFC and amygdala cannot be conclusively determined on the basis of PPI alone (Friston et al., 1997; Banks et al., 2007), vmPFC inhibition of the amygdala is of considerable significance for translational neuroscience. Hypothetically, the vmPFC controls decrements in fear response and strengthens extinction memory formation (Quirk and Mueller, 2008). Non-human animal research has consistently demonstrated this top-down inhibition of the amygdala by the vmPFC during fear extinction (Morgan et al., 1993; Milad and Quirk, 2002; Quirk et al., 2003; Rosenkranz et al., 2003; Delgado et al., 2008). Verifying vmPFC inhibition of the amygdala in humans will require further development of fMRI-based causality models (Etkin et al., 2006; McFarlin et al., 2012). Using dynamic causal modeling to indicate directionality between these regions in humans, Etkin et al. (2006) were able to demonstrate that pregenual ACC activity (adjacent to the vmPFC activity found here) predicted reductions in amygdala activity when the previous trial was incongruent (more emotional conflict). Structural equation modeling (SEM) on timeseries data has provided further support for medial PFC regulation of the amygdala (Meyer-Lindenberg and Zink, 2007). Despite not directly assessing causality, the present study extends prior work documenting heightened amygdala responses in specific phobia (Etkin and Wager, 2007) by highlighting the importance of the vmPFC and its connectivity with the amygdala for both the development and treatment of specific phobic (Maier and Watkins, 2010).

Additionally, a growing number of studies have implicated the vmPFC in emotional functions other than regulation (Hartley et al., 2011; Myers-Schulz and Koenigs, 2012). More specifically, the vmPFC region found here corresponds to the perigenual vmPFC section described by Myers-Schulz and Koenigs (2012) to be involved in positive affect. The identification of this area provided further support for the hypothesis that this area is involved in the psychologically beneficial effects provided during the anticipation of behavioral control over an aversive stimulus in phobics, perhaps related to down regulation of the amygdala and stress-related responses by the vmPFC.

Precisely because excessive anticipation of negative events has been shown to be maladaptive and contribute to psychiatric disorders (Mackiewicz et al., 2006; Nitschke et al., 2006, 2009; Straube et al., 2007; Sarinopoulos et al., 2010), this study investigated the neural basis for perceived control in humans to provide the proverbial “missing links” between learned helplessness (Seligman, 1975), social cognitive theory (Bandura, 2002), and the neuroscientific basis of resilience (Curtis and Cicchetti, 2003). As demonstrated by Seligman and colleagues, it is the perception of control that determines the behavioral response to a stressor. In particular, high resilience – the knowledge and prior experience of escapable shocks – reduced the learned helplessness behavior (Overmier and Seligman, 1967; Seligman and Maier, 1967). Furthermore, an animal’s resilience can be increased through behavioral treatment (Seligman et al., 1968). Thus, humans have an innate ability to change their capacity to perceive and exert control, in part due to their unique ability to attribute causality to aversive events that directly contribute to resilience (Abramson et al., 1978). The central role of perceived control in resilience (Staudinger et al., 1995; Chorpita and Barlow, 1998; Kumpfer, 1999; Maier et al., 2006) in conjunction with findings here indicate a prominent role for the vmPFC in the neurobiology of resilience. Coupling the neural circuitry for perceived control in humans with the underappreciated potential of human resilience points to the necessity of neuroscience in designing studies to enhance resilience in the face of adversity (Garmezy, 1971; Masten, 2001, 2011; Huber and Mathy, 2002; Casey, 2011).

### Limitations

Signal dropout was observed in a number of subjects in the vmPFC as is commonly reported in other studies. In the present study, the dropout extended into our functionally defined ROI in nine subjects, which led them to be excluded from the PPI analysis. Although the smaller sample limits the generalizability of the PPI results, it is unlikely that excluding these subjects biased the results because the extent of signal dropout was not associated with differences with demographic or psychological variables. Moreover, reducing the sample limits the statistical power for detecting the hypothesized effect here, lending support for the importance of the functional coupling found in the small sample. Another limitation is that we were not able to test whether the effects observed here for anticipation were also present for the video presentation. Analogous analyses for the video period were not possible due to insufficient trials per cell: only half the trials included a video as a result of the experimental manipulation of controllability.

## Conclusion

This first study of behavioral control investigating anticipatory responses directly extends Maier’s model of behavioral control to humans. The anticipatory vmPFC activation observed for perceived control has ramifications for the emotional response to aversive events and consequent effects of emotion and cognitive function. A new advance in functional connectivity, gPPI (McLaren et al., 2012), provided evidence of the dynamic relationships between nodes of the network, in particular the vmPFC and amygdala. The identification in humans of these brain areas in perceived controllability under aversive conditions clearly suggests that resilience is not only ordinary (Masten, 2001), but innate and potentially universal. As such, a neurological mechanism has evolved in humans to enable coping with extreme adversity, whether natural or social, and to perceive the controllability of our environment and emotional responses.

## Conflict of Interest Statement

The authors declare that the research was conducted in the absence of any commercial or financial relationships that could be construed as a potential conflict of interest.

## Supplementary Material

The Supplementary Material for this article can be found online at http://www.frontiersin.org/Emotion_Science/10.3389/fpsyg.2012.00557/abstract

## Footnotes

^1^http://www.nitrc.org/projects/peak_nii

^2^http://www.nitrc.org/projects/gppi
